# Book Reviews

**Published:** 1913-01

**Authors:** 


### BOOK REVIEWS

### UNITED STATES CIVIL SERVICE EXAMINATION

### PHYSICIAN (*Male*).—FEBRUARY 5, 1913.

The United States Civil Service Commission announces an open competitive examination for physician, for men only, on February 5 1913, at the places mentioned in the list printed here. on. From the eligibles resulting from this examination certification will be made to fill a vacancy in the Indian Service at each of the following places, and vacancies as they may occur in positions requiring similar qualifications in any branch of the service. unless it is found to be in the interest of the service to fill any vacancy by reinstatement, transfer, or promotion:

Navajo Agency (Tohatchi Boarding School), $1,000 per annum.

Cheyenne River Agency, South Dakota, $1,000 per annum.

Santee Agency, Nebraska, $1,000 per annum;

Western Navajo Agency, Arizona, $1,200 per annum.

SURGICAL AFTER-TREATMENT. By L. R. G. Crandon, M. D., Assistant in Surgery at Harvard Medical School; and Albert Ehrenfried, M. D., Assistant in Anatomy at Harvard Medical School. Octavo of 859 pages, with 290 original illustrations. M. B. Saunders & Co., Philadelphia.

This work tells how best to manage all problems and emergencies of surgical convalescence from recovery-room to discharge. It gives all the details completely, definitely, yet concisely, and does not refer the reader to some other work perhaps not then available.

---

OPERATIVE OBSTETRICS: Including Surgery of the New-born. By Edward P. Davis, M. D., Professor of Obstetrics in the Jefferson Medical College. Octavo of 483 pages, with 264 illustrations. Cloth, $5.50 net; Half Morocco, $7.00 net. W. B. Saunders & Co., Philadelphia.

The progress in obstetric surgery during the past few years has been remarkable. Operative measures in childbirth have taken great strides, and to-day every practitioner must be in a position to resort to the use of these modern methods in certain cases—and to-day's case may be such a case.

Mr. Davis in this volume gives all these new methods, all those operative procedures that you, any moment, may be called upon to use. And he gives them in such a clear, straightforward, definte way that you are able at once to grasp the meaning and follow the technic precisely.

